# Modifiable Perioperative Practices for the Prevention of Postoperative Complications After Cardiac Surgery: A Narrative Review

**DOI:** 10.3390/medicina62081469

**Published:** 2026-07-29

**Authors:** Livia Gheța, Oana Pătru, Mirela Vîrtosu, Andrei Grigorescu, Laurențiu Brăescu, Gemil Alsarhan, Darius Buriman, Horea Feier

**Affiliations:** 1 Doctoral School, “Victor Babes” University of Medicine and Pharmacy, 300041 Timisoara, Romania; livia.laurentiu@umft.ro (L.G.); daniela.cozma@umft.ro (M.V.); darius.buriman@umft.ro (D.B.); 2 Institute of Cardiovascular Diseases Timisoara, 13A Gheorghe Adam Street, 300310 Timisoara, Romania; grigorescu.andrei@umft.ro (A.G.); braescu.laurentiu@umft.ro (L.B.); alsarhangemil@gmail.com (G.A.); horea.feier@umft.ro (H.F.); 3 Research Center of the Institute of Cardiovascular Diseases Timisoara, 13A Gheorghe Adam Street, 300310 Timisoara, Romania; 4 Department of Cardiology, “Victor Babes” University of Medicine and Pharmacy, 2 Eftimie Murgu Sq., 300041 Timisoara, Romania; 5 Centre for Translational Research and Systems Medicine, “Victor Babes” University of Medicine and Pharmacy, 2 Eftimie Murgu Sq., 300041 Timisoara, Romania; 6 Department of Functional Sciences, Discipline of Pathophysiology, “Victor Babes” University of Medicine and Pharmacy, 2 Eftimie Murgu Sq., 300041 Timisoara, Romania

**Keywords:** cardiac surgery, perioperative monitoring, cardiothoracic critical care, postoperative complications, surgical site infection, patient safety, infection prevention, multidisciplinary care, organizational factors

## Abstract

*Background and Objectives*: Despite substantial advances in surgical techniques, anesthesia, and perioperative care, postoperative complications remain a major source of morbidity, mortality, prolonged hospitalization, and healthcare utilization following adult cardiac surgery (CS). Increasing evidence suggests that many of these complications are influenced by modifiable perioperative factors that can be addressed through multidisciplinary care. *Materials and Methods*: A narrative review was conducted to synthesize current evidence regarding perioperative practices associated with the prevention of postoperative complications in adult CS. A comprehensive literature search of PubMed/MEDLINE, Scopus, and Web of Science identified studies published between January 2015 and April 2026, supplemented by landmark studies and relevant clinical guidelines. *Results*: The identified evidence was organized into four major domains: infection prevention practices, physiological optimization strategies, protocol adherence and patient safety measures, and organizational and human factors. The strongest evidence supports timely antimicrobial prophylaxis, standardized infection prevention bundles, perioperative glycemic control, maintenance of normothermia, and patient blood management as key interventions associated with improved postoperative outcomes. Surgical safety checklists, standardized perioperative pathways, and adherence to evidence-based protocols further contributed to improved patient safety and consistency of care. Emerging evidence also highlighted the importance of communication, teamwork, safety culture, workload management, and healthcare professionals’ knowledge in facilitating successful implementation of perioperative interventions. *Conclusions*: Prevention of postoperative complications following CS requires a multidisciplinary, systems-based approach integrating evidence-based clinical interventions with standardized perioperative protocols and effective organizational practices that facilitate consistent implementation of evidence-based perioperative care. Future research should focus on prospective evaluation of integrated perioperative strategies, development of practical risk-stratification models, and further investigation of organizational determinants influencing implementation and postoperative outcomes.

## 1. Introduction

Cardiac surgery (CS) remains one of the most complex areas of contemporary surgical practice and continues to be associated with a substantial burden of postoperative complications despite major advances in surgical techniques, anesthesia, perioperative monitoring, and intensive care management. Surgical site infections (SSIs), deep sternal wound infections (DSWIs), postoperative bleeding, prolonged mechanical ventilation, extended intensive care unit (ICU) stay, and mortality remain major contributors to patient morbidity, healthcare utilization, and treatment costs. Despite continuous improvements in perioperative care, postoperative complications remain a significant challenge across the continuum of cardiac surgical management, particularly among patients with multiple comorbidities and increased clinical vulnerability [1,2].

The development of postoperative complications is multifactorial and reflects the interaction between patient-related characteristics, procedural complexity, and perioperative care processes. While non-modifiable risk factors, including advanced age, diabetes mellitus, obesity, impaired cardiac function, and pre-existing comorbidities, have been extensively investigated, increasing attention has focused on identifying modifiable perioperative factors that may improve postoperative outcomes. Emerging evidence further suggests that the cumulative burden of clinical vulnerability factors may substantially increase the risk of adverse postoperative events and healthcare-associated infections (HAIs) [3,4,5].

The perioperative period represents a critical opportunity for implementing preventive strategies. Evidence-based interventions, including timely antimicrobial prophylaxis, optimized perioperative glycemic control, maintenance of normothermia, patient blood management, standardized skin antisepsis, and surgical safety checklists, have been associated with improved postoperative outcomes and enhanced patient safety. Successful implementation of these interventions requires coordinated participation from the multidisciplinary perioperative team, including surgeons, anesthesiologists, perfusionists, nurses, residents, and support personnel [1,6,7,8].

Beyond technical and clinical interventions, evidence highlights the influence of organizational and human factors on perioperative performance and patient outcomes. Communication, teamwork, workload, fatigue, protocol adherence, safety culture, and staffing patterns may influence the consistency with which evidence-based practices are implemented in routine clinical care. The effectiveness of perioperative interventions depends not only on the quality of the interventions themselves but also on the organizational environment in which they are delivered [9,10,11,12,13].

Although studies have evaluated individual perioperative interventions and specific postoperative complications, the available evidence remains fragmented across multiple clinical domains. Most published reviews focus on isolated aspects of perioperative care, whereas relatively few provide a comprehensive synthesis of modifiable perioperative practices that can be implemented by multidisciplinary teams to improve outcomes following adult CS [1,14].

Therefore, the aim of this review is to identify and synthesize current evidence regarding modifiable perioperative practices associated with the prevention of postoperative complications in adult patients undergoing CS. To provide a structured overview of this broad field, the available evidence was organized into four complementary domains: infection prevention practices, physiological optimization strategies, patient safety and protocol adherence, and organizational and human factors. These domains were selected because they encompass the principal modifiable components of perioperative care that can be influenced by multidisciplinary teams and collectively contribute to postoperative outcomes following CS. Although presented separately for clarity, these domains are closely interconnected in clinical practice and should be considered complementary components of comprehensive perioperative care. Unlike recent clinical practice guidelines and consensus statements, which primarily focus on individual perioperative interventions and procedure-specific recommendations, this narrative review adopts an integrative perspective by synthesizing evidence on both clinical and organizational determinants of postoperative outcomes. In addition to evidence-based perioperative interventions, we examine the influence of teamwork, communication, workload, safety culture, protocol adherence, and healthcare professionals’ knowledge, attitudes, and practices, highlighting how these complementary factors interact to support safer and more effective perioperative care in adult CS.

## 2. Materials and Methods

This narrative review was conducted to identify and synthesize current evidence regarding modifiable perioperative practices associated with the prevention of postoperative complications following adult CS. A narrative review approach was considered the most appropriate methodology because the objective was to provide an integrated synthesis of evidence across multiple complementary domains of perioperative care, including clinical interventions, patient safety measures, and organizational and human factors, rather than to answer a single narrowly focused clinical question. The review primarily focused on studies published between January 2015 and April 2026. Earlier landmark studies and influential clinical guidelines were additionally included when considered essential for understanding the development and contemporary implementation of perioperative practices in CS.

A comprehensive literature search was performed using PubMed/MEDLINE, Scopus, and Web of Science. The search strategy combined Medical Subject Headings (MeSH) and free-text terms related to CS, postoperative complications, perioperative care, patient safety, infection prevention, protocol adherence, and multidisciplinary perioperative practice. Search terms included combinations of “cardiac surgery”, “cardiovascular surgery”, “coronary artery bypass grafting”, “valve surgery”, “postoperative complications”, “surgical site infection”, “sternal wound infection”, “mediastinitis”, “perioperative care”, “antibiotic prophylaxis”, “normothermia”, “skin preparation”, “sterile technique”, “surgical safety checklist”, “patient safety”, “compliance”, “teamwork”, and “workload”. Additional relevant publications were identified through manual screening of the reference lists of eligible articles.

Studies were considered eligible if they involved adult patients undergoing CS and evaluated perioperative interventions, clinical practices, patient safety measures, or organizational factors potentially associated with postoperative outcomes. Eligible study designs included randomized controlled trials, prospective and retrospective cohort studies, observational studies, quasi-experimental studies, quality-improvement initiatives, systematic reviews, narrative reviews and clinical guidelines. Because high-level evidence regarding organizational and human factors remains limited in CS, scoping reviews, perspective articles, consensus papers, and studies conducted in broader surgical settings were also considered when they provided clinically relevant insights into multidisciplinary perioperative practice. Preference was given to studies evaluating teamwork, communication, workload, safety culture, and other organizational factors in complex perioperative environments with potential applicability to cardiac surgical care. Studies focusing exclusively on pediatric populations, surgical techniques without a perioperative care component, animal studies, editorials, letters, and expert opinion papers were excluded.

Relevant publications were reviewed and data were extracted regarding study design, patient population, cardiac procedures, perioperative practices, healthcare professionals involved, postoperative outcomes, and principal findings. For the purpose of evidence synthesis, perioperative practices were categorized into four thematic domains: (1) infection prevention, (2) physiological optimization, (3) patient safety and protocol adherence, and (4) organizational and human factors. Representative studies and evidence sources underpinning the narrative synthesis are summarized in Supplementary Table S1.

The review specifically focused on interventions that could be implemented or influenced by members of the multidisciplinary perioperative team, including surgeons, anesthesiologists, perfusionists, nurses, residents, and support personnel. Outcomes of interest included surgical site infection, deep sternal wound infection, mediastinitis, postoperative bleeding, prolonged mechanical ventilation, prolonged intensive care unit stay, reintervention, and mortality.

Given the heterogeneity of study designs, interventions, and outcome measures, findings were synthesized narratively across the four predefined thematic domains. When formulating the overall conclusions, greater emphasis was placed on evidence derived from clinical practice guidelines, randomized controlled trials, and systematic reviews, while findings from observational studies were interpreted in the context of their methodological limitations. The objective of the review was to summarize and critically interpret current evidence regarding modifiable perioperative practices associated with improved postoperative outcomes and to identify areas requiring further clinical investigation.

## 3. Results

The literature identified four principal domains of modifiable perioperative practices associated with postoperative outcomes following adult CS: infection prevention, physiological optimization, patient safety and protocol adherence, and organizational and human factors. Infection prevention and physiological optimization represented the most extensively investigated areas, whereas more recent studies have increasingly focused on protocol adherence, multidisciplinary teamwork, and organizational factors that influence the implementation of evidence-based perioperative care [1,11,14,15]. Across these domains, the most frequently evaluated outcomes included SSIs, DSWIs, mediastinitis, prolonged ICU stay, prolonged mechanical ventilation, reintervention, and mortality.

The following sections summarize the evidence identified within each of these four thematic domains.

Table 1 and Table 2 summarizes the evidence included in this review.

### 3.1. Infection Prevention Practices

Infection prevention represented the most extensively investigated domain identified in the reviewed literature. SSIs, including superficial and DSWIs, remain among the most clinically significant complications following CS because of their association with prolonged hospitalization, reintervention, increased healthcare costs, and mortality [1,14]. Current evidence indicates that preventive strategies achieve the greatest benefit when implemented as multimodal perioperative interventions rather than as isolated measures [3,14].

Several perioperative practices have been evaluated for their role in reducing postoperative infectious complications. The most frequently investigated interventions include antimicrobial prophylaxis, perioperative skin antisepsis, maintenance of sterile technique, infection prevention bundles, and postoperative wound management. Collectively, the available evidence supports a multidisciplinary, bundle-based approach integrating complementary evidence-based interventions throughout the perioperative pathway [3,14,16].

#### 3.1.1. Antibiotic Prophylaxis

Appropriate antimicrobial prophylaxis remains one of the most consistently recommended and extensively investigated strategies for preventing SSIs following CS. Both the timing of antibiotic administration and adherence to established prophylactic protocols are recognized as key determinants of postoperative infectious outcomes.

Administration of prophylactic antibiotics within the recommended interval before surgical incision is associated with lower rates of SSI by ensuring adequate tissue antimicrobial concentrations at the time of incision. Delayed administration or administration performed excessively early has been associated with an increased risk of postoperative infection, supporting current recommendations regarding optimal timing and intraoperative redosing during prolonged procedures or substantial blood loss [7,16,42]. Beyond the timing of administration before surgical incision, appropriate antimicrobial prophylaxis also includes weight-based dosing, intraoperative redosing during prolonged procedures or major blood loss, and limiting postoperative prophylaxis according to current guideline recommendations. These measures help maintain adequate tissue antibiotic concentrations while minimizing unnecessary antimicrobial exposure [7,42,43,44].

Evidence specific to CS further supports the importance of adequate perioperative antibiotic exposure. Appropriate cefazolin tissue concentrations have been associated with lower rates of sternal surgical site infection, emphasizing that both antibiotic selection and maintenance of adequate antimicrobial concentrations throughout surgery contribute to effective infection prevention [43].

Antimicrobial prophylaxis is also a fundamental component of multimodal infection prevention bundles. Contemporary perioperative strategies integrate appropriate antibiotic prophylaxis with glycemic control, maintenance of normothermia, standardized skin antisepsis, and other evidence-based preventive measures. Although the contribution of individual bundle components may vary, available evidence suggests that multimodal approaches provide greater benefit than isolated interventions [3,14].

Overall, current clinical practice guidelines and high-quality evidence from SRs consistently support timely and protocol-compliant antimicrobial prophylaxis as a fundamental component of infection prevention in adult CS. Observational studies further reinforce these recommendations by demonstrating lower SSI rates when prophylactic antibiotics are administered according to established protocols.

#### 3.1.2. Skin Preparation and Sterile Technique

Preoperative skin antisepsis and maintenance of sterile technique are fundamental components of perioperative infection prevention in CS, reducing the risk of SSIs, DSWIs, and other postoperative infectious complications.

Among skin antiseptic agents, chlorhexidine- and povidone-iodine-based alcohol formulations have been the most extensively investigated. Earlier evidence suggested a potential advantage of chlorhexidine-alcohol preparations over povidone-iodine in reducing SSIs, particularly in clean surgical procedures [45]. However, more recent randomized clinical trials conducted in cardiac and major surgery populations have not demonstrated a clear superiority of one antiseptic agent over the other, indicating that both alcohol-based chlorhexidine and povidone-iodine preparations provide comparable protection when applied within standardized perioperative protocols [46,47]. Contemporary systematic reviews similarly suggest that alcohol-based antiseptic formulations may offer an overall benefit compared with aqueous preparations, although heterogeneity among studies limits definitive conclusions regarding the optimal antiseptic agent for CS [48].

Hair removal represents another modifiable component of perioperative infection prevention. Current evidence does not support routine preoperative hair removal unless it is required to facilitate the surgical procedure. When hair removal is necessary, clipping is preferred over razor shaving because it causes less skin trauma and has been consistently associated with a lower risk of postoperative surgical site infection [49].

Maintenance of sterile technique throughout the perioperative period remains equally important. Although the independent contribution of individual aseptic practices to SSI reduction is difficult to quantify, adherence to sterile principles, including appropriate surgical hand antisepsis, sterile gowning and gloving, maintenance of instrument sterility, and prompt correction of breaches in aseptic technique, is universally incorporated into contemporary infection prevention bundles and perioperative safety protocols. The effectiveness of these measures depends largely on consistent protocol adherence by the entire multidisciplinary surgical team [1,14].

Overall, evidence from RCTs and SRs supports standardized skin preparation protocols, avoidance of unnecessary hair removal, and the use of clipping when hair removal is required. However, the comparative effectiveness of individual antiseptic agents remains uncertain, and current evidence suggests that consistent adherence to standardized protocols may be more important than the choice of a specific antiseptic solution.

#### 3.1.3. Infection Prevention Bundles

Over the past decade, increasing attention has been directed toward multimodal infection prevention bundles as an effective strategy for reducing SSIs following CS [14,50]. Rather than relying on individual preventive measures, these bundles integrate complementary evidence-based interventions targeting modifiable perioperative risk factors throughout the preoperative, intraoperative, and postoperative phases of care [3,14,16].

Successful infection prevention depends on the coordinated implementation of multiple interventions rather than on any single preventive measure. Common bundle components include timely antimicrobial prophylaxis, preoperative skin decolonization, perioperative glycemic control, maintenance of normothermia, standardized skin antisepsis, adherence to sterile technique, and structured postoperative wound surveillance [3,14].

Evidence from observational studies in CS indicates that implementation of standardized infection prevention bundles is associated with lower postoperative infection rates and improved adherence to evidence-based perioperative practices. However, patient-related factors such as diabetes mellitus, obesity, perioperative hyperglycemia, and chronic respiratory disease continue to influence residual infection risk, emphasizing that bundle implementation should complement, rather than replace, individualized perioperative risk assessment [3,16].

Quality-improvement initiatives have further demonstrated that sustained reductions in deep sternal wound infections can be achieved through multidisciplinary infection prevention programs combining standardized perioperative protocols, continuous surveillance, performance monitoring, and active engagement of surgeons, anesthesiologists, nurses, and other members of the perioperative team. Long-term implementation of these strategies has also been associated with durable reductions in postoperative infection rates, highlighting the importance of protocol standardization and institutional commitment to continuous quality improvement [50].

Although the composition of infection prevention bundles varies considerably across institutions and published studies, evidence from recent systematic reviews consistently supports multimodal perioperative strategies over isolated preventive interventions. Nevertheless, differences in bundle composition, implementation methods, and outcome definitions limit direct comparisons between studies and preclude identification of a single optimal bundle. Overall, the available evidence suggests that consistent protocol adherence and multidisciplinary collaboration are more important determinants of successful infection prevention than the specific combination of interventions included in an individual bundle [14].

#### 3.1.4. Postoperative Wound Management

Postoperative wound management represents an important component of infection prevention following CS. Although considerable attention has traditionally focused on intraoperative preventive measures, increasing evidence suggests that postoperative wound care, surveillance, and early recognition of complications may also influence patient outcomes.

DSWIs and mediastinitis remain among the most serious postoperative complications after CS because of their association with prolonged hospitalization, reintervention, increased healthcare costs, and mortality. Reported rates of sternal wound infection generally range between 1% and 5%, while severe infections may be associated with mortality exceeding 10% in selected patient populations [51,52].

Several studies have evaluated the role of postoperative dressing strategies in reducing SSIs and sternal wound complications. Current evidence suggests that conventional dressings, absorbent dressings, and negative-pressure wound therapy (NPWT) provide comparable overall SSI rates, although NPWT may offer additional benefit in carefully selected high-risk patients, particularly those undergoing coronary artery bypass grafting (CABG) with internal mammary artery grafts [53].

Recent evidence syntheses similarly indicate that prophylactic NPWT may reduce postoperative wound complications in selected high-risk patients; however, the overall certainty of evidence remains moderate, and further well-designed randomized studies are required before routine implementation can be universally recommended [14].

Beyond dressing selection, structured postoperative wound surveillance has emerged as an essential component of comprehensive infection prevention programs. Early recognition of wound erythema, drainage, dehiscence, fever, or sternal instability facilitates timely intervention and may prevent progression to DSWIs or mediastinitis. Consequently, standardized postoperative wound assessment protocols and multidisciplinary follow-up have become integral components of contemporary cardiac surgical care [1,53].

Overall, the available evidence supports postoperative wound management as an extension of perioperative infection prevention rather than an isolated postoperative intervention. While evidence supporting routine use of advanced dressing strategies remains limited to selected high-risk populations, structured wound surveillance, early recognition of complications, and timely intervention are consistently recommended as key components of comprehensive SSI prevention following CS.

Collectively, current evidence, including clinical practice guidelines, SRs, and RCTs, supports a multimodal approach to infection prevention in CS. Timely antimicrobial prophylaxis, standardized skin antisepsis, multimodal infection prevention bundles, and structured postoperative wound surveillance represent the most consistently supported strategies. Nevertheless, the effectiveness of these interventions appears to depend largely on their consistent implementation within standardized perioperative care pathways and multidisciplinary clinical practice.

### 3.2. Physiological Optimization Strategies

Physiological optimization represents a fundamental component of perioperative care in CS. In addition to infection prevention measures, several modifiable physiological factors have been associated with postoperative outcomes, including body temperature regulation, glycemic control, and perioperative blood management. Unlike many other surgical specialties, CS frequently requires cardiopulmonary bypass (CPB), which may induce hemodilution, activation of inflammatory and coagulation pathways, metabolic disturbances, and ischemia–reperfusion injury, thereby increasing the risk of postoperative complications. Consequently, maintenance of physiological homeostasis throughout the perioperative period has become a central objective of contemporary cardiac surgical care [54,55].

Alterations in body temperature, glucose homeostasis, coagulation, and tissue oxygen delivery may contribute to impaired wound healing, SSIs, acute kidney injury, prolonged ICU stay, and increased postoperative morbidity. Accordingly, contemporary clinical guidelines advocate integrated perioperative strategies aimed at maintaining physiological stability, minimizing preventable organ dysfunction, and optimizing postoperative recovery [6,17].

The available evidence has primarily focused on three major components of physiological optimization: prevention of perioperative hypothermia, optimization of perioperative glycemic control, and implementation of patient blood management (PBM) strategies. Although the strength of evidence differs across these interventions, contemporary clinical practice guidelines consistently recommend their integration into perioperative care pathways as key components of multidisciplinary management involving surgeons, anesthesiologists, perfusionists, intensivists, and nursing staff [6,17,18].

#### 3.2.1. Temperature Management and Prevention of Hypothermia

Maintenance of perioperative normothermia is considered a fundamental component of physiological optimization in CS [1,18]. Inadvertent perioperative hypothermia, commonly defined as a core body temperature below 36 °C, frequently occurs during major surgical procedures because of anesthesia-induced impairment of thermoregulation, exposure of body surfaces, administration of unwarmed intravenous fluids, and operating room environmental conditions [19,20].

Several physiological mechanisms have been proposed to explain the association between hypothermia and adverse postoperative outcomes. Reduced body temperature may promote peripheral vasoconstriction, impair tissue oxygenation, alter neutrophil function, disrupt coagulation pathways, and delay wound healing, thereby potentially increasing susceptibility to SSIs, perioperative bleeding, and delayed postoperative recovery [19].

Evidence accumulated over the past decades supports the routine use of perioperative temperature monitoring and active warming strategies to maintain normothermia. Landmark randomized trials demonstrated reductions in SSIs and length of hospital stay with active temperature management, findings that have subsequently been incorporated into contemporary perioperative guidelines [17,19].

More recent systematic reviews have confirmed that active warming strategies effectively maintain perioperative normothermia and improve thermal stability throughout surgery. However, the relationship between hypothermia and SSIs remains less consistent. Although severe hypothermia and temperatures below 35 °C appear to increase infection risk, recent meta-analyses have reported substantial heterogeneity across surgical populations, operative procedures, and temperature thresholds, limiting definitive conclusions regarding the magnitude of this association [20,21].

Perioperative hypothermia may adversely influence postoperative recovery. Hypothermia at ICU admission has been independently associated with prolonged ICU stay following elective CABG, supporting the importance of maintaining adequate temperature control throughout both the intraoperative and early postoperative periods [22].

Beyond infection prevention, maintenance of normothermia may also improve perioperative hemostasis. Active warming has been associated with reduced intraoperative blood loss compared with passive warming strategies, further supporting its incorporation into standardized perioperative care pathways [23].

Overall, routine perioperative temperature monitoring and active warming are integral components of physiological optimization in CS. Although the direct relationship between hypothermia and SSIs remains incompletely defined, maintenance of normothermia appears to contribute to improved physiological stability, reduced blood loss, and enhanced postoperative recovery.

#### 3.2.2. Perioperative Glycemic Control

Perioperative glycemic control has emerged as one of the most important modifiable determinants of postoperative outcomes in CS [8,24]. Hyperglycemia commonly develops during and after cardiac procedures as a consequence of surgical stress, cardiopulmonary bypass, systemic inflammatory responses, and pre-existing metabolic disorders. Importantly, perioperative dysglycemia affects both patients with diabetes mellitus and those without a previous diagnosis of diabetes [24,54].

Current evidence consistently associates uncontrolled perioperative hyperglycemia with increased rates of SSIs, impaired wound healing, prolonged ICU stay, and greater postoperative morbidity. Hyperglycemia may impair neutrophil function, reduce tissue oxygenation, and compromise host immune responses, thereby increasing susceptibility to postoperative infectious complications. Consequently, perioperative glycemic management has become a central component of enhanced recovery pathways and contemporary cardiac surgical care [17,24].

Repeated postoperative hyperglycemic episodes are associated with higher rates of infectious complications, stroke, and mortality. Furthermore, intensive perioperative glycemic control has been associated with reduced rates of postoperative infections and selected cardiac complications, particularly DSWIs and postoperative AF, although consistent reductions in mortality have not been demonstrated across all studies [8,25,26].

Current recommendations emphasize the importance of balancing the benefits of glycemic control against the risk of hypoglycemia. Accordingly, most contemporary guidelines recommend maintaining blood glucose concentrations between 140 and 180 mg/dL in the majority of cardiac surgical patients, avoiding both severe hyperglycemia and excessively intensive glucose-lowering strategies. Careful glucose monitoring and individualized insulin therapy are therefore essential to minimize glycemic variability while preventing hypoglycemic episodes, both of which may adversely affect postoperative outcomes [17,24].

Beyond absolute glucose values, increasing evidence suggests that glycemic variability represents an independent predictor of adverse postoperative outcomes. Greater perioperative glucose variability has been associated with increased short- and long-term mortality following CS, highlighting the importance of continuous glucose monitoring and stable glycemic management throughout the perioperative period [27].

Overall, perioperative glycemic control is a key component of physiological optimization in CS. Although the optimal glucose target remains an area of ongoing investigation, avoidance of significant hyperglycemia and excessive glycemic variability appears to be consistently associated with improved postoperative outcomes.

#### 3.2.3. Blood Transfusion and Patient Blood Management

Perioperative bleeding and blood transfusion remain major determinants of postoperative outcomes following CS. Owing to the complexity of cardiac procedures, the use of CPB, and procedure-related coagulation disturbances, patients undergoing CS are among the largest consumers of allogeneic blood products. However, blood transfusion is not a benign intervention and may itself contribute to postoperative morbidity. Allogeneic transfusion has been associated with increased rates of postoperative infection, acute kidney injury, prolonged hospitalization, and mortality, prompting widespread adoption of patient blood management strategies aimed at minimizing unnecessary transfusions while maintaining adequate tissue oxygen delivery and hemostasis [6].

PBM is a multidisciplinary, evidence-based approach encompassing preoperative anemia screening and treatment, optimization of hemoglobin concentration, intraoperative blood conservation techniques, individualized coagulation management, and restrictive transfusion strategies. The primary objective is to reduce exposure to allogeneic blood products while improving clinical outcomes and patient safety. In addition to transfusion practices, preoperative anemia has emerged as an important modifiable risk factor. Lower preoperative hemoglobin concentrations have been associated with an increased risk of anemia-related postoperative complications, supporting systematic identification and optimization of anemic patients before CS [6,28,29].

Evidence from CS consistently demonstrates that structured PBM programs reduce transfusion requirements while supporting improved postoperative outcomes. These programs typically combine standardized transfusion algorithms, point-of-care coagulation testing, blood conservation techniques, and enhanced perioperative monitoring. By reducing unnecessary exposure to allogeneic blood products, PBM programs may decrease postoperative complications while also reducing healthcare resource utilization [6,30].

The association between blood transfusion and adverse postoperative outcomes has been consistently reported across the literature. Although part of this relationship reflects greater baseline clinical complexity and operative risk among transfused patients, contemporary evidence supports a restrictive, individualized transfusion strategy whenever clinically appropriate. Accordingly, modern perioperative guidelines advocate protocolized management of perioperative bleeding based on coagulation monitoring and multidisciplinary decision-making to optimize hemostasis while minimizing unnecessary blood product exposure [6].

Contemporary PBM encompasses several complementary strategies, including preoperative identification and treatment of anemia, judicious use of antifibrinolytic agents, intraoperative cell salvage when appropriate, viscoelastic-guided coagulation management to support targeted transfusion therapy, and restrictive transfusion thresholds. Integrating these measures into standardized perioperative care pathways may reduce unnecessary transfusions while maintaining patient safety [6,30].

Overall, PBM is a cornerstone of physiological optimization in CS. Through systematic identification and treatment of preoperative anemia, optimization of coagulation management, and implementation of evidence-based transfusion strategies, PBM programs may contribute to improved postoperative outcomes, enhanced patient safety, and more efficient utilization of healthcare resources.

### 3.3. Protocol Adherence and Patient Safety Measures

In recent years, increasing attention has been directed toward the role of protocol adherence and patient safety initiatives in improving perioperative outcomes following CS. Although evidence-based interventions such as antimicrobial prophylaxis, glycemic control, maintenance of normothermia, and PBM are well established, their effectiveness depends largely on consistent implementation throughout the perioperative pathway by the multidisciplinary surgical team [1,15,31].

Variability in clinical practice, incomplete adherence to standardized protocols, communication failures, and omission of critical safety steps have all been recognized as potentially modifiable contributors to preventable adverse events. Consequently, contemporary cardiac surgical practice increasingly emphasizes standardized perioperative pathways, structured safety protocols, and continuous quality-improvement initiatives aimed at reducing unwarranted variation in care delivery and improving postoperative outcomes [1,18].

Among the most widely implemented patient safety interventions are structured surgical safety checklists, standardized perioperative protocols, and compliance-monitoring strategies designed to improve the reliability of evidence-based care. These interventions are complemented by multidisciplinary communication processes and continuous performance evaluation, which collectively support safer and more consistent perioperative practice [31,32].

#### 3.3.1. Surgical Safety Checklists and Prevention of Perioperative Errors

Structured surgical safety checklists represent one of the most widely implemented patient safety interventions in contemporary perioperative practice. Introduced through the World Health Organization (WHO) Safe Surgery Saves Lives initiative, the Surgical Safety Checklist was developed to standardize critical perioperative processes, improve multidisciplinary communication, and reduce preventable surgical complications [32,33].

The checklist comprises three sequential phases—Sign In, Time Out, and Sign Out—performed before induction of anesthesia, before skin incision, and before the patient leaves the operating room. These structured checkpoints facilitate communication among surgeons, anesthesiologists, nurses, perfusionists, and other members of the perioperative team while ensuring that essential safety measures are completed consistently [32,34].

Implementation of surgical safety checklists is associated with reductions in postoperative complications, adverse events, and mortality across a broad range of surgical specialties. Beyond verification of procedural steps, checklists promote structured discussion of patient-specific risks, anticipated intraoperative challenges, equipment availability, sterility concerns, and postoperative management plans, thereby strengthening team communication and improving the reliability of perioperative care [32].

These benefits appear particularly relevant in CS, where procedures are characterized by prolonged operative times, CPB, advanced technology, and close interaction among multiple healthcare professionals. Consequently, professional societies such as the European Association for Cardio-Thoracic Surgery (EACTS) recommend incorporation of structured safety checklists into routine perioperative practice as part of broader quality-improvement initiatives.

Successful checklist implementation, however, depends not only on the existence of the checklist itself but also on meaningful team engagement and consistent protocol adherence. Inadequate training, incomplete checklist execution, poor staff engagement, and variable participation among team members may substantially reduce its effectiveness. These observations emphasize that surgical safety checklists should be viewed as communication and teamwork tools rather than simple documentation requirements [31].

In addition to reducing common postoperative complications, standardized safety practices may contribute to the prevention of rare but catastrophic perioperative adverse events. Analysis of “never events” following adult CS demonstrated that, although uncommon, these events were associated with substantial morbidity, prolonged hospitalization, and increased healthcare costs. Most were considered potentially preventable through rigorous protocol adherence, structured multidisciplinary communication, and institutional quality assurance processes, further reinforcing the importance of standardized patient safety measures in contemporary cardiac surgical practice [35].

Overall, surgical safety checklists are an effective component of perioperative patient safety programs. Their clinical impact depends not only on checklist availability but also on consistent implementation, multidisciplinary engagement, and integration into a broader culture of patient safety.

#### 3.3.2. Compliance with Perioperative Protocols

The effectiveness of evidence-based perioperative interventions depends not only on the availability of standardized protocols but also on their consistent implementation in routine clinical practice. Variability in protocol adherence has been recognized as an important contributor to preventable adverse events, particularly in complex surgical environments such as CS. Increasing attention has been directed toward identifying factors that influence compliance with perioperative recommendations and their impact on patient outcomes [15,31].

Higher adherence to perioperative protocols is associated with improved patient outcomes, including lower postoperative complication rates, reduced mortality, and fewer unplanned ICU admissions. Successful implementation strategies commonly incorporate audit and feedback, staff education, reminders, multidisciplinary engagement, and continuous performance monitoring, emphasizing that implementation strategies are as important as the protocols themselves [31].

Also, protocol adherence should be viewed as more than completion of mandatory documentation. High-quality compliance, characterized by active multidisciplinary participation, effective communication, and meaningful engagement during perioperative safety processes, has been associated with greater improvements in patient safety than superficial or ritualized completion of protocols [36].

Organizational support also appears to play a critical role in sustaining adherence to evidence-based perioperative practice. Leadership commitment, continuous staff education, regular performance feedback, and institutional monitoring systems have been consistently identified as important facilitators of long-term protocol implementation and quality-improvement initiatives [15].

In addition to organizational factors, the KAP of healthcare professionals may directly influence protocol adherence. Evidence from CS settings has identified gaps in familiarity with perioperative guidelines despite generally positive attitudes toward evidence-based practice, suggesting that ongoing education and reinforcement of best-practice recommendations remain essential for maintaining high-quality perioperative care [37].

Overall, protocol adherence is a critical determinant of perioperative patient safety. Consistent implementation of evidence-based protocols requires multidisciplinary engagement, organizational support, continuous education, and a positive safety culture capable of sustaining high-quality perioperative practice.

#### 3.3.3. Standardization of Care Pathways

Standardization of perioperative care pathways has emerged as an important strategy for reducing variability in clinical practice and improving outcomes following CS [1,19]. Structured perioperative pathways aim to ensure that evidence-based interventions are delivered consistently throughout the surgical continuum, thereby minimizing omissions, delays, and unwarranted variation in clinical practice [15,31].

Enhanced Recovery After Surgery (ERAS) principles and other protocolized perioperative pathways have been increasingly adapted to CS. These multidisciplinary programs integrate multiple evidence-based interventions, including preoperative optimization, standardized anesthetic management, perioperative glycemic control, maintenance of normothermia, PBM, infection prevention measures, early mobilization, and coordinated postoperative care. By incorporating these components into a unified care pathway, ERAS programs seek to improve both process reliability and clinical outcomes [1].

Implementation of standardized cardiac surgical pathways is associated with shorter hospital and ICU LOS, reduced postoperative opioid requirements, and enhanced postoperative recovery without increasing complication rates. Although the overall certainty of evidence remains limited by the relatively small number of randomized controlled trials, current findings support protocolized perioperative pathways as a safe and effective approach to optimizing recovery after CS [18].

Beyond their direct clinical effects, standardized care pathways appear to strengthen multidisciplinary communication and coordination throughout the perioperative process. Clearly defined protocols facilitate role clarification, establish shared expectations, and promote consistent implementation of evidence-based practices among surgeons, anesthesiologists, perfusionists, nurses, and intensive care teams. These organizational benefits may be particularly relevant in CS, where patient management requires close collaboration across multiple professional disciplines [1].

The effectiveness of standardized care pathways, however, depends on successful local implementation and sustained protocol adherence. Contemporary quality-improvement initiatives consistently demonstrate that protocol availability alone is insufficient to ensure compliance. Continuous education, performance monitoring, audit and feedback, and institutional leadership remain essential for achieving long-term implementation and maintaining improvements in perioperative care [15,31].

Overall, the available evidence supports standardized perioperative care pathways as an effective strategy for reducing practice variability and facilitating consistent delivery of evidence-based interventions. Their greatest benefit appears to be achieved when protocolized care is combined with multidisciplinary collaboration, continuous professional education, and robust quality-improvement processes.

### 3.4. Organizational and Human Factors

Beyond clinical interventions and standardized perioperative care pathways, increasing attention has been directed toward the influence of organizational and human factors on patient safety in CS [15,34]. The operating room represents a complex sociotechnical environment in which clinical outcomes depend not only on technical expertise but also on effective communication, teamwork, leadership, situational awareness, workload management, and organizational culture [10,11].

CS is particularly susceptible to human-factor-related challenges because of procedural complexity, prolonged operative duration, CPB, multidisciplinary team involvement, and the need for continuous coordination among surgeons, anesthesiologists, perfusionists, nurses, intensivists, and support personnel. Emerging evidence suggests that optimization of these non-technical skills, together with the development of a positive safety culture, may contribute to improved perioperative performance and safer patient care [9,15].

#### 3.4.1. Team Communication and Teamwork

Effective communication and teamwork are widely recognized as fundamental determinants of patient safety in the cardiac operating room [11,34]. Cardiac surgical procedures require continuous coordination among surgeons, anesthesiologists, perfusionists, nurses, and other healthcare professionals, making effective multidisciplinary collaboration essential for safe and efficient perioperative care [9,38].

The American Heart Association scientific statement on patient safety in the cardiac operating room identified communication failures, teamwork deficiencies, workflow disruptions, and ineffective handovers as important contributors to preventable adverse events. Accordingly, structured communication protocols, multidisciplinary briefings, standardized handoff procedures, and surgical safety checklists have been recommended to strengthen team performance and improve patient safety [34].

Subsequent evidence has reinforced these recommendations. A scoping review by Ghanmi et al. identified communication-focused interventions, clearly defined professional roles, structured teamwork practices, and reduction in intraoperative distractions as key strategies associated with improved team performance in the operating room [11]. Similarly, recent multicenter evidence from CS demonstrated that greater familiarity among members of the operative team was associated with improved safety and procedural efficiency, highlighting the importance of stable multidisciplinary teams in high-complexity surgical environments [9].

Beyond communication itself, psychological safety has emerged as an important determinant of effective team functioning. Team members who feel comfortable speaking up, questioning decisions, and reporting potential safety concerns appear more likely to contribute to safer perioperative processes, particularly in highly hierarchical surgical environments [39]. In parallel, increasing attention has been directed toward objective assessment of both technical and non-technical skills in CS, reflecting growing recognition that communication, leadership, situational awareness, and teamwork represent measurable components of surgical quality and patient safety [40].

The importance of the collaborative relationship between surgeons and perfusionists during CS should be emphasized. Strengthening the perfusionist–surgeon partnership, promoting shared decision-making, and fostering a culture of psychological safety have been proposed as practical strategies for reducing preventable errors and enhancing patient safety in the cardiac operating room [38].

Overall, it is suggested that effective multidisciplinary communication, stable team dynamics, psychological safety, and structured collaboration represent important non-technical components of perioperative patient safety. Although their direct impact on postoperative clinical outcomes requires further investigation, these factors appear to facilitate consistent implementation of evidence-based perioperative practices and may complement technical interventions aimed at reducing preventable adverse events.

#### 3.4.2. Workload, Fatigue and Burnout

Workload, fatigue, and burnout have emerged as important organizational factors that may influence both healthcare professionals’ well-being and patient safety in perioperative environments [12,13]. CS is characterized by prolonged procedures, high cognitive demands, time pressure, and complex multidisciplinary interactions, all of which may contribute to increased workload and occupational stress among perioperative personnel [13,41].

Excessive workload may adversely affect team performance, communication, situational awareness, and clinical decision-making. High workload levels are frequently reported among members of perioperative teams and have been identified as potential contributors to performance deterioration, supporting the incorporation of workload assessment into patient safety and quality-improvement initiatives [13].

Burnout has likewise emerged as an important concern in perioperative practice. Recent evidence indicates consistently high levels of emotional exhaustion, depersonalization, and reduced professional accomplishment among surgeons, anesthesiologists, and operating room nurses. These factors may negatively influence communication, teamwork, workflow efficiency, and overall patient safety [12].

Studies involving perioperative nurses further suggest that higher burnout levels are associated with reduced job performance and increased exposure to patient safety risks. Workplace disorganization, negative team climate, and excessive workload have been identified as important contributors to burnout, emphasizing the close relationship between organizational conditions and the quality of perioperative care [56].

Fatigue has also been recognized as a significant threat to patient safety because of its potential effects on concentration, situational awareness, communication, and clinical decision-making [12]. Accordingly, contemporary patient safety frameworks increasingly advocate organizational strategies aimed at identifying and mitigating fatigue-related risks through appropriate staffing, workload management, and supportive workplace policies. Beyond individual well-being, broader organizational factors, including staffing levels, workload distribution, leadership support, and workplace culture, appear to influence teamwork, safety perceptions, and adherence to evidence-based perioperative practices. Positive organizational environments have been consistently associated with stronger safety culture and improved implementation of patient safety initiatives [10,15].

Overall, workload, fatigue, and burnout should be regarded as important organizational determinants of perioperative patient safety. Although direct evidence linking these factors to specific postoperative complications in CS remains limited, their influence on communication, teamwork, protocol adherence, and overall clinical performance supports their inclusion in contemporary patient safety and quality-improvement frameworks.

#### 3.4.3. Knowledge, Attitudes and Practices of Perioperative Personnel

Successful implementation of evidence-based perioperative interventions ultimately depends on the healthcare professionals responsible for delivering care. Although clinical guidelines, infection prevention bundles, and patient safety protocols are widely available, differences in healthcare professionals’ knowledge, attitudes, and routine clinical practices may substantially influence their implementation and overall effectiveness [15,37].

Greater familiarity with evidence-based recommendations is associated with improved compliance with perioperative protocols, more consistent implementation of infection prevention measures, and stronger engagement in patient safety initiatives. Conversely, knowledge gaps and barriers to guideline implementation may contribute to variability in clinical practice despite the availability of well-established recommendations [37].

Attitudes toward evidence-based practice also appear to influence implementation success. Healthcare professionals who perceive clinical guidelines as relevant, feasible, and beneficial are generally more likely to incorporate recommended interventions into routine practice. Likewise, positive attitudes toward patient safety have been associated with greater participation in multidisciplinary quality-improvement initiatives and sustained implementation of evidence-based perioperative care [15].

Beyond knowledge and attitudes, organizational constraints, including workload, staffing shortages, limited resources, and workplace culture, may influence the translation of evidence into everyday clinical practice. These findings emphasize that successful implementation depends not only on individual competence but also on supportive organizational environments that facilitate adherence to best-practice recommendations [12].

Collectively, the available evidence indicates that assessment healthcare professionals’ knowledge, attitudes, and practices (KAP) may help identify barriers to implementation and opportunities for targeted educational interventions. Continuous professional development, regular training, and reinforcement of evidence-based recommendations may strengthen protocol adherence, promote a positive safety culture, and support consistent delivery of high-quality perioperative care.

Overall, healthcare professionals’ KAP represent important determinants of perioperative care quality. Although direct evidence linking KAP interventions to postoperative outcomes in CS remains limited, optimization of these factors may facilitate implementation of evidence-based perioperative practices and complement broader multidisciplinary patient safety initiatives.

**Figure 1:**
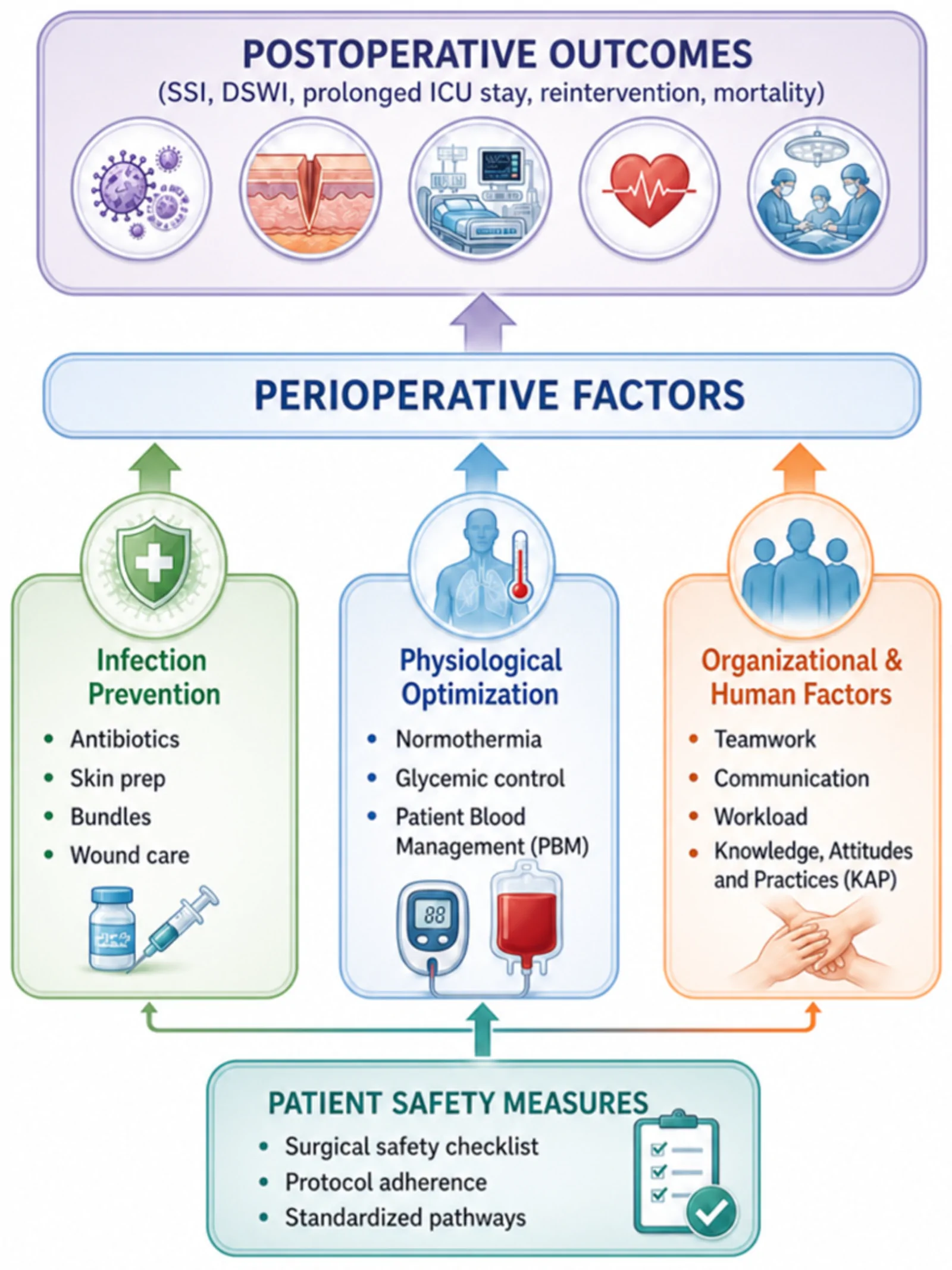
Integrated conceptual framework summarizing the four principal domains of modifiable perioperative factors associated with postoperative outcomes following adult CS. The framework illustrates the interaction between infection prevention, physiological optimization, patient safety and protocol adherence, and organizational and human factors within the multidisciplinary perioperative pathway.

The evidence identified in this review suggests that postoperative outcomes following CS are influenced by four major domains of modifiable perioperative factors. Figure 1 summarizes the conceptual framework derived from the reviewed literature.

## 4. Discussion

### 4.1. Main Findings

CS remains associated with a substantial risk of postoperative complications despite major advances in surgical techniques and perioperative care. The findings of this review suggest that postoperative outcomes are influenced by a complex interaction between infection prevention measures, physiological optimization strategies, patient safety practices, and organizational factors. The available evidence indicates that successful perioperative management depends not on isolated interventions but on coordinated multimodal approaches implemented throughout the entire perioperative pathway.

Among the domains identified, infection prevention demonstrated the most consistent body of evidence. Timely antimicrobial prophylaxis, standardized infection prevention bundles, perioperative skin antisepsis, and structured postoperative wound surveillance were consistently associated with improved outcomes and lower rates of SSIs. These findings support the concept that infection prevention should be regarded as a continuous perioperative process extending from preoperative preparation through postoperative recovery rather than as a single intervention performed immediately before surgery [7,14,16].

### 4.2. Clinical Implications for Perioperative and Intensive Care Practice

The findings of this review have several practical implications for multidisciplinary teams involved in CS. Many of the perioperative factors identified in the present review are potentially modifiable and therefore represent realistic targets for quality-improvement initiatives. Improving postoperative outcomes depends not only on the availability of evidence-based interventions but also on their consistent implementation across the entire perioperative pathway [1,2,15].

Among infection-prevention interventions, timely administration of antimicrobial prophylaxis remains one of the most consistently supported measures. Appropriate antibiotic selection, administration before surgical incision, and intraoperative redosing during prolonged procedures are essential components of effective SSI prevention programs. However, isolated interventions are unlikely to achieve optimal results when implemented independently. Instead, multimodal infection-prevention bundles incorporating antimicrobial prophylaxis, standardized skin antisepsis, glycemic control, maintenance of normothermia, and structured postoperative wound surveillance appear to provide the greatest clinical benefit [3,7,14].

Similarly, physiological optimization emerged as a cornerstone of contemporary perioperative care. Perioperative hyperglycemia, hypothermia, preoperative anemia, and unnecessary exposure to allogeneic blood transfusion have all been associated with adverse postoperative outcomes. Consequently, strategies aimed at maintaining physiological homeostasis should be regarded as fundamental components of contemporary cardiac surgical pathways. The consistent benefits reported for perioperative glycemic control, active warming strategies, and PBM support their integration into routine clinical practice [6,8,17].

Many of the perioperative interventions identified in this review continue to influence patient outcomes beyond the operating room and therefore require ongoing optimization during the postoperative ICU stay. Glycemic control, temperature management, wound surveillance, transfusion practices, and early recognition of postoperative complications remain key components of postoperative care that may substantially influence recovery following CS. Recent evidence further highlights the importance of structured multidisciplinary management within specialized cardiac ICUs, where timely recognition of complications and prompt decision-making, including when reoperation is required, may contribute to improved postoperative outcomes and more efficient resource utilization [6,8,56,57].

Beyond prevention of infectious complications, perioperative physiological optimization should also be viewed as a strategy for preserving end-organ function throughout the postoperative period. Acute kidney injury and neurological complications remain among the most clinically relevant adverse events following CS and contribute substantially to prolonged hospitalization, functional impairment, and mortality. Contemporary clinical practice guidelines therefore advocate integrated perioperative and intensive care strategies focused on maintaining physiological stability, optimizing tissue perfusion, and minimizing avoidable organ dysfunction through coordinated multidisciplinary care [17,58].

Beyond postoperative monitoring, structured nursing interventions have been associated with improved recovery of cardiac function, better postoperative quality of life, and enhanced patient engagement, emphasizing the role of nurses in patient education, behavioral support, early mobilization, postoperative surveillance, and continuity of care throughout the perioperative continuum [59]. Successful implementation may be as important as the intervention itself. Protocol adherence, standardized care pathways, and surgical safety checklists have consistently been associated with improved perioperative outcomes, suggesting that variability in clinical practice remains an underrecognized contributor to preventable postoperative complications. Healthcare institutions should not only develop evidence-based protocols but also establish mechanisms for monitoring compliance, providing continuous feedback, and supporting sustainable implementation of best practices [15,31,32].

Contemporary CS increasingly advocates integration of validated risk prediction models into routine perioperative decision-making to support individualized preventive strategies and optimize postoperative outcomes. In addition to established clinical risk scores, comprehensive preoperative assessment, including multimodality cardiovascular imaging when appropriate, may improve characterization of complex cardiac pathology and facilitate personalized surgical planning, particularly in patients with congenital or structurally complex heart disease [60,61,62].

From a practical perspective, the evidence synthesized in this review suggests that prevention of postoperative complications should be viewed as a shared multidisciplinary responsibility involving surgeons, anesthesiologists, intensivists, perfusionists, nurses, residents, and support personnel. The effectiveness of perioperative interventions ultimately depends not only on the quality of individual clinical interventions but also on their coordinated implementation across the entire perioperative and postoperative care pathway. Future quality-improvement initiatives should therefore combine evidence-based clinical interventions with standardized protocols, multidisciplinary education, effective communication, continuous outcome monitoring, and individualized risk stratification to support consistent delivery of high-quality perioperative and intensive care.

Based on the evidence identified in the present review, several practical recommendations can be proposed for multidisciplinary perioperative teams involved in cardiac surgical care (Table 3).

### 4.3. Organizational and Human Factors: Beyond Technical Interventions

Although infection prevention measures and physiological optimization strategies demonstrated the strongest clinical evidence, the findings of this review indicate that their effectiveness ultimately depends on successful implementation within complex perioperative environments. Postoperative outcomes are influenced not only by the interventions performed but also by the consistency, reliability, and quality with which they are implemented throughout the perioperative pathway.

CS represents one of the most demanding clinical environments, requiring continuous coordination among surgeons, anesthesiologists, perfusionists, intensivists, nurses, residents, and support personnel. Within these highly complex settings, communication failures, teamwork deficiencies, excessive workload, and organizational constraints may compromise protocol adherence and reduce the effectiveness of otherwise evidence-based interventions. Increasing evidence suggests that non-technical skills, including communication, leadership, situational awareness, and teamwork, are essential for maintaining safe perioperative care and supporting consistent implementation of patient safety practices [9,11,15].

Organizational factors influence not only individual clinical performance but also the successful implementation of standardized perioperative pathways. Surgical safety checklists, infection prevention bundles, and protocolized care pathways are unlikely to achieve their intended benefits unless supported by a positive safety culture, effective multidisciplinary communication, and sustained institutional commitment to quality improvement. These observations reinforce the concept that implementation strategies are as important as the clinical interventions themselves [10,31,32].

Importantly, organizational and human factors are unlikely to influence postoperative outcomes in isolation. Rather, they appear to determine the reliability with which evidence-based perioperative interventions are implemented in routine clinical practice. Effective teamwork, leadership, structured communication, and psychological safety facilitate consistent execution of antimicrobial prophylaxis, infection prevention bundles, glycemic control protocols, surgical safety checklists, and PBM strategies. Conversely, communication failures, unclear role allocation, and poor multidisciplinary coordination may reduce protocol adherence, increase process variability, and diminish the effectiveness of interventions that are otherwise supported by strong clinical evidence, thereby increasing the risk of preventable postoperative complications [9,10,11,15,31,32].

Workload, fatigue, and burnout represent additional organizational challenges with potential implications for perioperative patient safety. High cognitive demands, prolonged procedures, staffing limitations, and time pressure may adversely affect concentration, communication, decision-making, and overall team performance. Although direct evidence linking these factors to postoperative complications in CS remains limited, available studies consistently suggest that organizational strategies promoting adequate staffing, professional well-being, and supportive working environments are likely to strengthen patient safety and facilitate implementation of evidence-based perioperative care. These observations suggest that supporting healthcare professionals’ well-being should be regarded not only as a workforce priority but also as a patient safety strategy aimed at maintaining reliable perioperative performance [12,13].

Another important observation of this review is the central role of healthcare professionals in translating evidence into routine clinical practice. Appropriate antimicrobial prophylaxis, glycemic control, maintenance of normothermia, PBM, sterile technique, and postoperative wound surveillance all require coordinated action by multidisciplinary teams. Differences in healthcare professionals’ KAP may contribute to variability in protocol adherence and influence the effectiveness of otherwise well-established perioperative interventions. Accordingly, continuing education, simulation-based training, and regular audit and feedback may represent important implementation strategies for improving adherence to evidence-based perioperative practices [11,31,32]. This systems perspective suggests that optimization of perioperative outcomes requires simultaneous attention to both technical interventions and the organizational conditions that enable their reliable implementation.

Taken together, these findings support a systems-based approach to perioperative quality improvement. Organizational and human factors should therefore be regarded as facilitators of successful implementation rather than as separate components of perioperative care. Future patient safety initiatives should integrate evidence-based clinical protocols with implementation strategies aimed at strengthening teamwork, communication, leadership, professional education, psychological safety, and organizational culture across multidisciplinary perioperative and intensive care teams [9,10,11,12,13,14,15,31,32].

Finally, the relative scarcity of CS-specific studies evaluating organizational and human factors remains an important gap in the current literature. Future prospective, multicenter investigations are needed to clarify the relationship between non-technical skills, organizational performance, protocol adherence, and clinically relevant postoperative outcomes in CS. In particular, future studies should evaluate whether interventions targeting teamwork, leadership, communication, implementation strategies, and safety culture translate into measurable improvements in protocol adherence and patient outcomes.

### 4.4. Future Research Directions

Important knowledge gaps remain regarding the prevention of postoperative complications following CS. Most available studies have evaluated individual interventions or isolated risk factors, whereas postoperative outcomes are more likely determined by complex interactions among patient characteristics, perioperative management strategies, and organizational factors.

One of the principal limitations is the relative scarcity of prospective, multidisciplinary studies designed to evaluate multiple modifiable perioperative factors simultaneously. Although antimicrobial prophylaxis, glycemic control, maintenance of normothermia, and PBM have each demonstrated clinical benefits, considerably less is known about how these interventions interact when implemented within routine perioperative practice. Future investigations should therefore adopt integrated study designs capable of evaluating the combined effects of clinical, procedural, and organizational variables on postoperative outcomes.

Future research should focus on developing pragmatic perioperative risk-stratification models that integrate patient characteristics, intraoperative variables, postoperative physiological parameters, and healthcare-related exposures. Such multidimensional models may improve identification of patients at increased risk of postoperative complications and facilitate individualized preventive strategies, more efficient allocation of perioperative resources, and personalized postoperative surveillance [62].

This review also highlights the need for further investigation of organizational and human factors in CS. Although communication, teamwork, workload, fatigue, safety culture, and non-technical skills are increasingly recognized as important determinants of perioperative performance, direct evidence linking these factors to specific postoperative outcomes remains limited. Well-designed prospective multicenter studies are needed to clarify the mechanisms through which organizational factors influence protocol adherence, implementation of evidence-based interventions, and clinically relevant postoperative outcomes. Future studies should also evaluate whether interventions targeting teamwork, leadership, communication, psychological safety, and implementation strategies translate into measurable improvements in protocol adherence and patient outcomes.

Particular attention should be directed toward healthcare professionals’ KAP regarding evidence-based perioperative care. Many of the interventions identified in this review, including antimicrobial prophylaxis, infection prevention measures, glycemic control, PBM, and perioperative safety protocols, depend heavily on consistent implementation by multidisciplinary teams. Future research should determine whether targeted educational interventions, implementation strategies, and improvements in safety culture can enhance protocol adherence and translate into measurable improvements in patient outcomes.

Finally, future research should move beyond evaluation of isolated clinical interventions and adopt a systems-based perspective integrating traditional clinical risk factors with organizational, behavioral, and implementation-related determinants of care quality. Such an approach may provide a more comprehensive understanding of postoperative complication development and support the design of multifaceted quality-improvement strategies tailored to the unique challenges of contemporary CS and postoperative intensive care. Greater integration of implementation science into perioperative research may further facilitate translation of evidence-based recommendations into routine clinical practice. Greater integration of implementation science, digital clinical decision-support systems, and emerging artificial intelligence applications into perioperative research may further facilitate translation of evidence-based recommendations into routine clinical practice and support continuous quality improvement.

### 4.5. Limitations

Several limitations of the present review should be acknowledged. First, the available literature was characterized by substantial heterogeneity regarding study design, patient populations, perioperative interventions, and outcome definitions. Variability in the reporting of SSIs, DSWIs, and other postoperative complications limited direct comparisons between studies and precluded quantitative synthesis of the available evidence.

Second, much of the available evidence originated from observational studies, quality-improvement initiatives, institutional protocols, and clinical guidelines rather than large multicenter randomized controlled trials. Consequently, residual confounding, institutional variability, and differences in local perioperative practices should be considered when interpreting the reported associations between perioperative interventions and postoperative outcomes. Furthermore, despite the predefined search strategy and eligibility criteria, the narrative design may have introduced a degree of selection bias in the identification and interpretation of the available evidence.

Third, although this review primarily focused on studies published between 2015 and 2026, several landmark studies and influential clinical guidelines published before this period were included because they continue to provide the scientific foundation for many contemporary perioperative practices in CS. While their inclusion strengthened the contextual background of the review, it also introduced a degree of temporal heterogeneity into the evidence base.

Another important limitation concerns the organizational and human factors domain. Compared with infection prevention and physiological optimization strategies, considerably fewer studies have directly evaluated the relationship between teamwork, communication, workload, burnout, healthcare professionals’ KAP outcomes in CS. Furthermore, part of the evidence in this domain was derived from broader surgical populations rather than exclusively from CS settings. Although these findings are likely applicable to other high-complexity surgical environments, their extrapolation to CS should be interpreted with appropriate caution.

In addition, no formal assessment of methodological quality or risk of bias was performed, as this review was designed as a narrative rather than a systematic review. Moreover, the substantial heterogeneity of study designs, interventions, patient populations, and outcome measures precluded quantitative evidence synthesis or meta-analysis. Accordingly, the findings should be interpreted as a comprehensive narrative synthesis of the current literature rather than as a quantitative assessment of intervention effectiveness.

Finally, interpretation and thematic categorization of the available evidence inevitably involved a degree of subjective judgment, representing an inherent limitation of narrative reviews. Nevertheless, this approach allowed integration of diverse clinical, organizational, and patient safety perspectives that would have been difficult to capture through a conventional systematic review focused on a single intervention. The present review therefore provides a comprehensive overview of modifiable perioperative factors while highlighting important priorities for future research and quality-improvement initiatives in CS.

## 5. Conclusions

Postoperative complications remain a major source of morbidity, prolonged hospitalization, and healthcare resource utilization following CS. The evidence synthesized in this review indicates that several modifiable perioperative factors may contribute to improved postoperative outcomes when implemented as part of coordinated multidisciplinary perioperative care.

Importantly, successful prevention of postoperative complications depends not only on the availability of evidence-based clinical interventions but also on their consistent implementation throughout the perioperative and postoperative intensive care continuum. Effective communication, teamwork, protocol adherence, safety culture, and organizational support appear to play a critical role in translating evidence into routine clinical practice and maximizing the benefits of established perioperative interventions.

Overall, the findings of this review support a multidisciplinary, patient-centered, and systems-based approach to perioperative and intensive care in CS. Integrating evidence-based clinical interventions with standardized care pathways, individualized risk stratification, and continuous quality-improvement initiatives represents a clinically sound strategy that may reduce preventable postoperative complications and improve patient outcomes. However, while several individual interventions are supported by direct evidence in CS, the effectiveness of integrated multimodal perioperative care pathways has not yet been consistently demonstrated in prospective studies specific to this population. Future research should prioritize prospective evaluation of integrated perioperative care models, validation of practical risk-stratification tools, and further investigation of organizational and human factors that influence the quality and consistency of perioperative care.

## Figures and Tables

**Table 1:** Overview of the evidence included in the review.

Domain	Main Perioperative Practices Evaluated	Predominant Study Designs	Principal Postoperative Outcomes	Representative References
Infection prevention	Antibiotic prophylaxis, skin antisepsis, infection prevention bundles, wound management	RCTs, SRs, meta-analyses, cohorts	SSI, DSWI, mediastinitis	de Jonge et al., 2017; Rogers et al., 2024; Qaddumi et al., 2025 [7,14,16]
Physiological optimization	Normothermia, glycemic control, PBM	RCTs, SRs, meta-analyses, guidelines	SSI, AF, ICU stay, transfusion	Jin et al., 2020; Casselman et al., 2025; [6,8]
Patient safety measures	Surgical safety checklists, protocol adherence, ERAS	SRs, cluster RCTs	Complications, mortality, LOS	Engelman et al., 2019; Grant et al., 2024; Martinez-Nicolas et al., 2024 [1,2,15]
Organizational & human factors	Teamwork, communication, workload, KAP	Scoping, observational, cross-sectional	Team performance, protocol adherence	Ghanmi et al., 2024; Bauer et al., 2024; Totonchilar et al., 2024 [9,11,13]

**Table 2:** Summary of modifiable perioperative practices associated with postoperative outcomes following CS.

Domain	Practice/Intervention	Main Outcomes	Overall Conclusion	Primary Supporting Evidence
Infection Prevention	Antibiotic prophylaxis	SSI	Appropriate timing and protocol adherence are associated with a reduced risk of SSI	Clinical practice guidelines; SRs
Infection Prevention	Skin antisepsis	SSI	Chlorhexidine- and povidone-iodine-based preparations demonstrate comparable efficacy in CS	RCTs; SRs
Infection Prevention	Infection prevention bundles	SSI, mediastinitis	Multimodal bundles have been associated with improved infection-related outcomes	Clinical practice guidelines; observational studies
Infection Prevention	Wound surveillance and postoperative wound management	DSWI, mediastinitis	Early detection and standardized wound monitoring may facilitate timely management and reduce progression of wound complications	Observational studies; quality-improvement initiatives
Physiological Optimization	Normothermia maintenance	SSI, ICU stay, blood loss	Maintenance of normothermia supports physiological stability and has been associated with improved postoperative recovery	Clinical practice guidelines; RCTs
Physiological Optimization	Glycemic control	SSI, DSWI, mortality, atrial fibrillation	Clinical practice guidelines and high-quality evidence support perioperative glycemic control	Clinical practice guidelines; RCTs; SRs
Physiological Optimization	Patient blood management	Morbidity, transfusion requirements, LOS	Protocolized patient blood management has been associated with reduced transfusion requirements and improved postoperative outcomes	Clinical practice guidelines; RCTs
Patient Safety Measures	Surgical safety checklist	Complications, mortality	Checklist implementation has been associated with improved team communication and perioperative safety	Observational studies; quality-improvement studies
Patient Safety Measures	Protocol adherence	Multiple postoperative outcomes	Higher protocol adherence may be associated with improved postoperative outcomes	Observational studies
Patient Safety Measures	Standardized care pathways (ERAS)	LOS, recovery, complications	Standardized perioperative pathways may reduce practice variability and facilitate evidence-based care	Clinical practice guidelines; SRs; observational studies
Organizational and Human Factors	Team communication and teamwork	Safety outcomes	Effective communication and teamwork are associated with safer perioperative care	Observational studies; narrative reviews
Organizational and Human Factors	Workload, fatigue, and burnout	Errors, safety incidents	Excessive workload and fatigue may adversely affect team performance and patient safety	Observational studies; narrative reviews
Organizational and Human Factors	Knowledge, attitudes, and practices (KAP)	Protocol adherence, implementation of best practices	Education and training may improve adherence to evidence-based perioperative practices	Observational studies; quality-improvement studies

The evidence summarized in this table is based on representative studies and guidelines cited throughout the manuscript [1,2,3,4,5,6,7,8,9,10,11,12,13,14,15,16,17,18,19,20,21,22,23,24,25,26,27,28,29,30,31,32,33,34,35,36,37,38,39,40,41].

**Table 3:** Practical recommendations for multidisciplinary perioperative teams in CS.

Domain	Recommendation	Primary Supporting Evidence
Antibiotic prophylaxis	Administer prophylactic antibiotics within the recommended interval before incision and ensure intraoperative redosing when indicated	Clinical practice guidelines; SRs
Infection prevention bundles	Implement multimodal SSI prevention bundles throughout the perioperative pathway	SRs; observational studies
Glycemic control	Avoid severe hyperglycemia and excessive glycemic variability	Clinical practice guidelines; meta-analyses
Normothermia	Maintain perioperative core temperature ≥ 36 °C	Clinical practice guidelines; RCTs
Patient blood management	Apply restrictive and protocolized transfusion strategies when appropriate	Clinical practice guidelines; SRs
Surgical safety checklist	Ensure multidisciplinary completion of surgical safety checklists	Clinical practice guidelines; observational studies
Protocol adherence	Monitor compliance with evidence-based perioperative protocols	Observational studies; quality-improvement initiatives
Team communication	Promote structured briefings and debriefings among perioperative team members	Observational studies; implementation research
Education and training	Support continuing education regarding infection prevention and patient safety practices	Observational studies; quality-improvement initiatives

## Data Availability

No new data were created or analyzed in this study. Data sharing is not applicable to this article.

## Supplementary Materials

The following supporting information can be downloaded at: https://www.mdpi.com/article/10.3390/medicina62081469/s1, Table S1: Characteristics of the studies included in the narrative evidence synthesis.

## Abbreviations

The following abbreviations are used in this manuscript:

AF	Atrial Fibrillation
CABG	Coronary Artery Bypass Grafting
CPB	Cardiopulmonary Bypass
CS	Cardiac Surgery
DSWI	Deep Sternal Wound Infection
EACTS	European Association for Cardio-Thoracic Surgery
ERAS	Enhanced Recovery After Surgery
HAI	Healthcare-Associated Infection
ICU	Intensive Care Unit
KAP	Knowledge, Attitudes, and Practices
LOS	Length of Stay
MeSH	Medical Subject Headings
NPWT	Negative-Pressure Wound Therapy
PBM	Patient Blood Management
RCT	Randomized Controlled Trial
SSI	Surgical Site Infection
WHO	World Health Organization
