# Supplementary material for: Modifiable Perioperative Practices for the Prevention of Postoperative Complications After Cardiac Surgery: A Narrative Review
Source: Medicina (Kaunas). 2026 Jul 29;62(8):1469. doi: 10.3390/medicina62081469 (PMC13515097; doi:10.3390/medicina62081469)
Supplement: Supplementary file 1 [file medicina-62-01469-s001.zip › medicina-4434089-supplementary.pdf]

Table S1. Representative studies and evidence sources underpinning the narrative synthesis.

Domain	Author (Year)	Study design	Population	Perioperative intervention / factor evaluated	Main outcome(s)	Principal findings
**Infection Prevention**	Morris et al. (2025)	Cohort study	Orthopedic and CS patients	Timing of antibiotic prophylaxis	SSI	Appropriate timing of antibiotic prophylaxis was associated with lower SSI rates.
	de Jonge et al. (2017)	SR and meta-analysis	54,552 surgical patients	Timing of antibiotic prophylaxis	SSI	Delayed or excessively early antibiotic administration increased the risk of SSI.
	Zelenitsky et al. (2018)	Prospective cohort study	Adult CS patients	Cefazolin prophylaxis	Sternal SSI	Adequate cefazolin exposure was associated with lower rates of sternal SSI.
	Andrade et al. (2019)	Observational study	CS patients	SSI prevention bundle	SSI	Implementation of a multimodal prevention bundle improved adherence to infection prevention practices.
	Rogers et al. (2024)	Cochrane SR	Adult CS patients	SSI prevention strategies	SSI	Multimodal SSI prevention strategies demonstrated the strongest evidence for reducing

					postoperative infections.
Boisson et al. (2024)	RCT	Adult CS patients	Chlorhexidine-alcohol versus povidone-iodine-alcohol	Surgical site infection	No significant difference was observed between the two antiseptic strategies.
Widmer et al. (2024)	RCT	Surgical patients	Skin antisepsis	SSI	Chlorhexidine- and povidone-iodine-based antiseptic preparations demonstrated comparable efficacy.
Yang et al. (2024)	SR and meta-analysis	Surgical patients	Skin antisepsis solutions	SSI	Alcohol-based antiseptic preparations were associated with lower SSI rates than aqueous preparations.
Tanner & Melen (2021)	Cochrane SR	Surgical patients	Hair removal methods	Surgical site infection	Hair clipping was associated with lower SSI risk than shaving.
Qaddumi et al. (2025)	Cohort study	CS patients	SSI prevention bundle	Surgical site infection	Bundle implementation reduced postoperative infection rates.
Conoscenti et al. (2025)	Cohort study	CS patients	Long-term infection prevention program	SSI	Long-term implementation of preventive measures maintained reduced SSI rates over a

						10-year period.
	Pérez-Granda et al. (2025)	RCT	CS patients	Postoperative dressing strategies	SSI, mediastinitis	NPWT benefited selected high-risk patients.
**Physiological Optimization**	Poveda et al. (2020)	SR and meta-analysis	Surgical patients	Active warming interventions	Normothermia, SSI	Active warming effectively maintained perioperative normothermia and reduced hypothermia-related complications.
	Wang et al. (2023)	Observational study	CABG patients	Perioperative hypothermia	ICU LOS	Perioperative hypothermia was associated with prolonged ICU stay.
	Chen et al. (2025)	Meta-analysis	Surgical patients	Perioperative hypothermia	SSI	Perioperative hypothermia was associated with an increased risk of SSI.
	Yi et al. (2018)	RCT	Major surgery patients	Intraoperative warming	Blood loss	Maintenance of normothermia reduced intraoperative blood loss.
	Järvelä et al. (2018)	Cohort study	CS patients	Postoperative hyperglycemia	Postoperative infection	Hyperglycemia was associated with increased postoperative infection risk.
	Jin et al. (2020)	SR and meta-analysis	CS patients	Glycemic control strategies	DSWI, atrial fibrillation	Improved perioperative glycemic control reduced

						selected postoperative complications.
	Yao et al. (2025)	Retrospective cohort study	CS patients	Glycemic variability	Mortality	Greater glycemic variability was associated with increased postoperative mortality.
	Kattou et al. (2022)	Retrospective cohort study	Adult CS patients	Preoperative anemia	Transfusion, postoperative complications	Low preoperative hemoglobin independently predicted anemia-related postoperative complications.
	Casselman et al. (2025)	Clinical guideline synthesis	Adult CS patients	PBM	Morbidity, transfusion	Contemporary guidelines support multidisciplinary PBM strategies to reduce unnecessary transfusions and improve perioperative outcomes.
	Rancati et al. (2021)	Narrative review	CS patients	PBM	Transfusion-related outcomes	The review supports implementation of multidisciplinary PBM strategies
**Patient Safety Measures**	Qaiser et al. (2024)	SR	Surgical patients	WHO Surgical Safety Checklist	Complications, mortality	Checklist implementation improved patient safety and reduced postoperative

						complications.
	Emond et al. (2022)	Cluster-randomized multicenter trial	Surgical patients	Guideline adherence	Mortality, complications	Greater adherence to evidence-based perioperative guidelines was associated with improved clinical outcomes.
	Zhang et al. (2022)	SR	Adult CS patients	Enhanced Recovery After Surgery (ERAS) pathways	LOS, recovery	ERAS pathways were associated with shorter hospital stay and improved postoperative recovery.
**Organizational and Human Factors**	Wahr et al. (2013)	American Heart Association Scientific Statement	Cardiac operating room teams	Team communication and patient safety	Safety processes	Structured communication, teamwork, and standardized handovers were recommended to improve patient safety.
	Ghanmi et al. (2024)	Scoping review	Operating room teams	Teamwork interventions	Team performance	Communication-focused interventions consistently improved teamwork processes in operating room settings.
	Vikan et al. (2024)	Qualitative study	Operating room professionals	Psychological safety	Team functioning	Psychological safety promoted speaking up and facilitated

					safer perioperative processes.
Bauer et al. (2024)	Multicenter observational study	CS teams	Operative team familiarity	Safety, procedural efficiency	Greater familiarity among operative team members was associated with improved safety and procedural efficiency.
Likosky et al. (2021)	Mixed-methods study protocol	CS teams	Assessment of technical and non-technical skills	Surgical quality	Proposed a standardized framework for evaluating technical and non-technical performance.
Shann & Sundt (2025)	Perspective article	CS teams	Perfusionist–surgeon collaboration	Safety culture	Advocated strengthening surgeon–perfusionist collaboration to promote patient safety and organizational culture.
Totonchilar et al. (2024)	Multicenter cross-sectional study	Surgical team members	Workload	Team performance	High workload negatively affected team performance.
Hueto Madrid et al. (2025)	Narrative review	Operating room personnel	Burnout	Patient safety	Reviewed evidence suggesting that burnout may negatively influence perioperative performance and patient safety.

Bong et al. (2025)	Cross-sectional study	Perioperative healthcare professionals	Safety culture	Patient safety perceptions	A positive safety culture was associated with more favorable perceptions of patient safety.
Balachandran et al. (2025)	Cross-sectional study	Cardiac nurses	Knowledge of perioperative guidelines	Protocol adherence	Knowledge gaps and implementation barriers regarding perioperative guidelines were identified among cardiac nurses.
